# Supplementary material for: Structural and Theoretical Study of Copper(II)-5-fluoro Uracil Acetate Coordination Compounds: Single-Crystal to Single-Crystal Transformation as Possible Humidity Sensor
Source: Polymers (Basel). 2023 Jun 26;15(13):2827. doi: 10.3390/polym15132827 (PMC10346346; doi:10.3390/polym15132827)
Supplement: Supplementary file 1 [file polymers-15-02827-s001.zip › polymers-2395563-supplementary.pdf]

Structural and Theoretical Study of Copper(II)-5-Fluoro Uracil Acetate Coordination Compounds: Single-Crystal to Single-Crystal Transformation as Possible Humidity Sensor

Verónica G. Vegas[1], Andrea García-Hernán [1], Fernando Aguilar-Galindo [2], Josefina Perles [3,*] and Pilar Amo-Ochoa [1,4,*]

[1] Dpto. de Química Inorgánica, Facultad de Ciencias, Universidad Autónoma de Madrid, 28049 Madrid, Spain; veronica.garciav@estudiante.uam.es (V.G.V.); andrea.garciah@uam.es (A.G.-H.)

[2] Dpto. de Química, Facultad de Ciencias, Universidad Autónoma de Madrid, 28049 Madrid, Spain;
fernando.aguilar-galindo@uam.es

[3] Laboratorio de DRX Monocristal, Servicio Interdepartamental de Investigación, Universidad Autónoma de Madrid, 28049 Madrid, Spain

[4] Institute for Advanced Research in Chemical Sciences (IAdChem), Universidad Autónoma de Madrid,
28049 Madrid, Spain

* Correspondence: josefina.perles@uam.es (J.P.); pilar.amo@uam.es (P.A.-O.)

S1. Crystals transformations

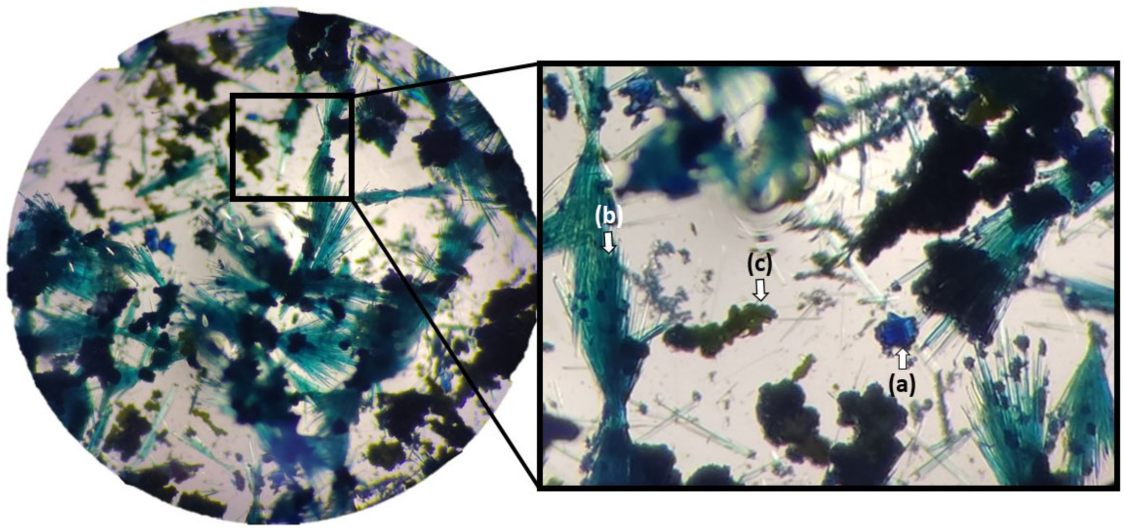

Figure S1. Microscope image (x2.0 zoom) of phases detected in the reactor at initial time in the hydrothermal reaction corresponding with: dark blue crystal **[Cu(5-FUA)$_2$(H$_2$O)(bipy)]$_n$·2nH$_2$O (CP3) (a)**, elongated turquoise crystals **[Cu$_2$(5-FUA)$_2$(ox)(bipy)]$_n$·2nH$_2$O (CP2) (b)**, and yellow crystal of **[(H$_2$bipy)$^{+2}$ 2.NO$_3^-$] (1) (c).**

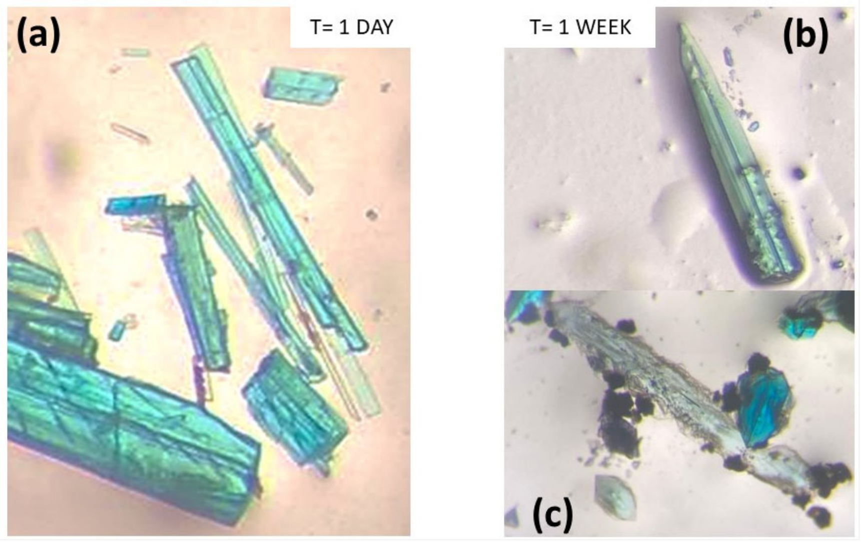

Figure S2. Crystal of **[Cu$_2$(5-FUA)$_2$(ox)(bipy)]$_n$·2nH$_2$O** (a) transformation to **[Cu(ox)(bipy)]$_n$** (b) and **[Cu$_3$(ox)$_3$(bipy)$_4$]$_n$** (c)

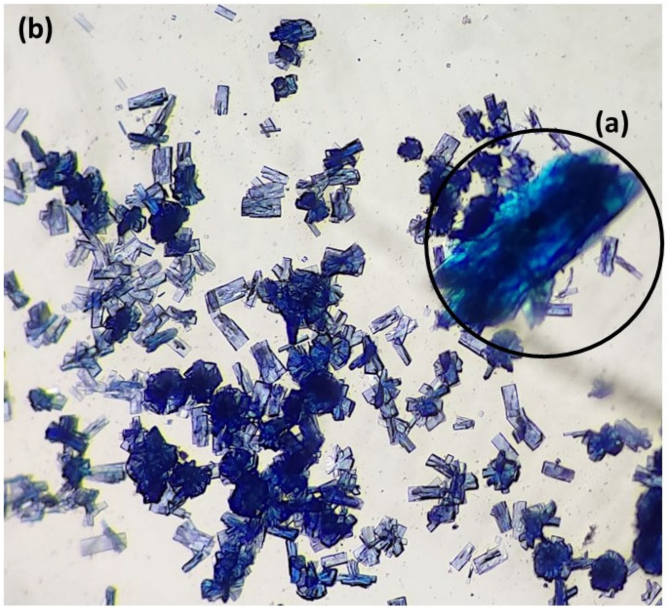

Figure S3. Microscope image (x4.0 zoom) of phases in the separated mother liquor: **[Cu(5-FUA)$_2$(H$_2$O)(bipy)]$_n$·2nH$_2$O (CP3)** (a) and **[Cu(5-FUA)$_2$(bipy)]$_n$·3.5nH$_2$O** (b).

S2. Chemical characterization of the compounds.

S2.1 FT-IR Spectrum

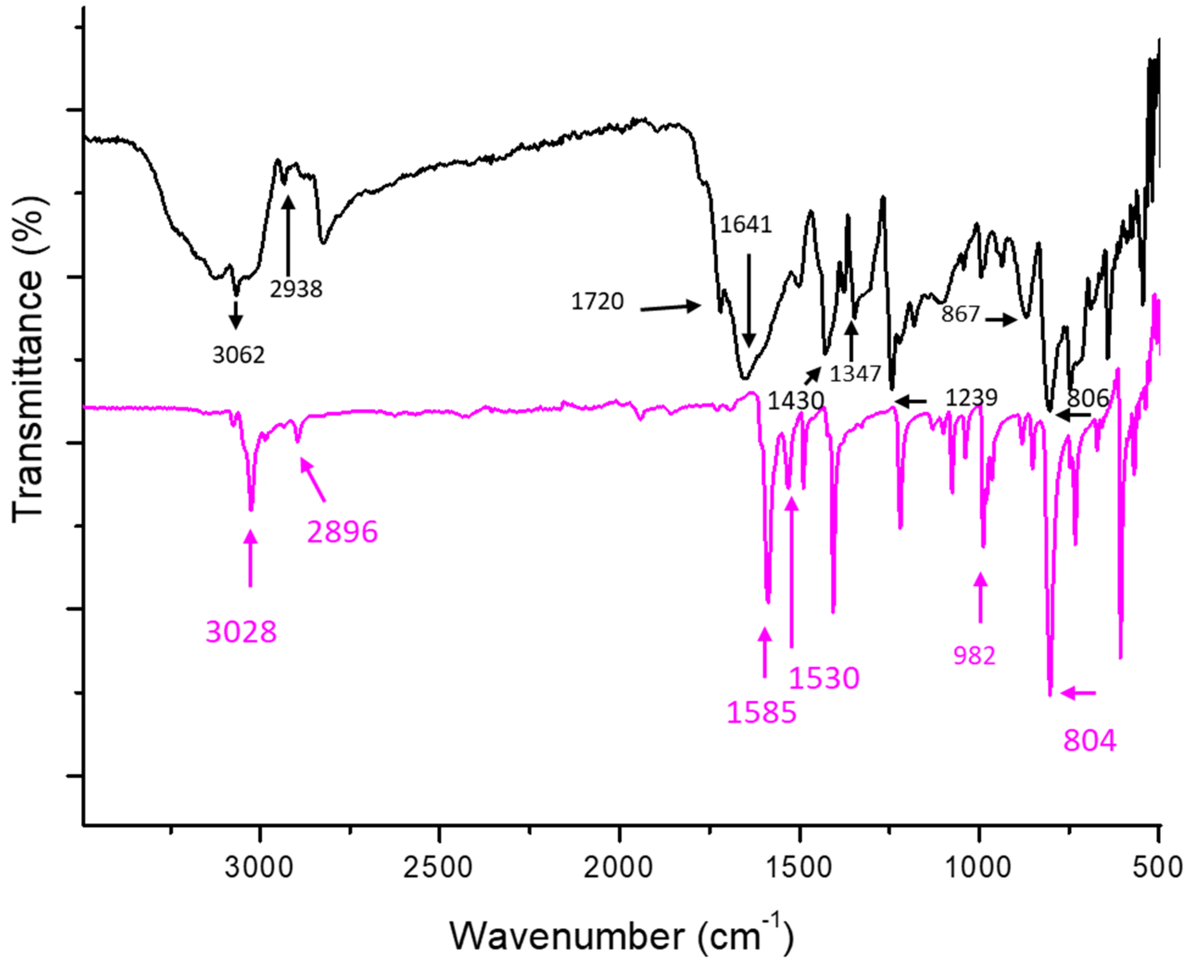

Figure S4. IR Spectra of **[(H$_2$bipy)$^{+2}$ 2.NO$_3^-$] (1)** black line and the ligand 4.4´-bipyridine in pink**.**

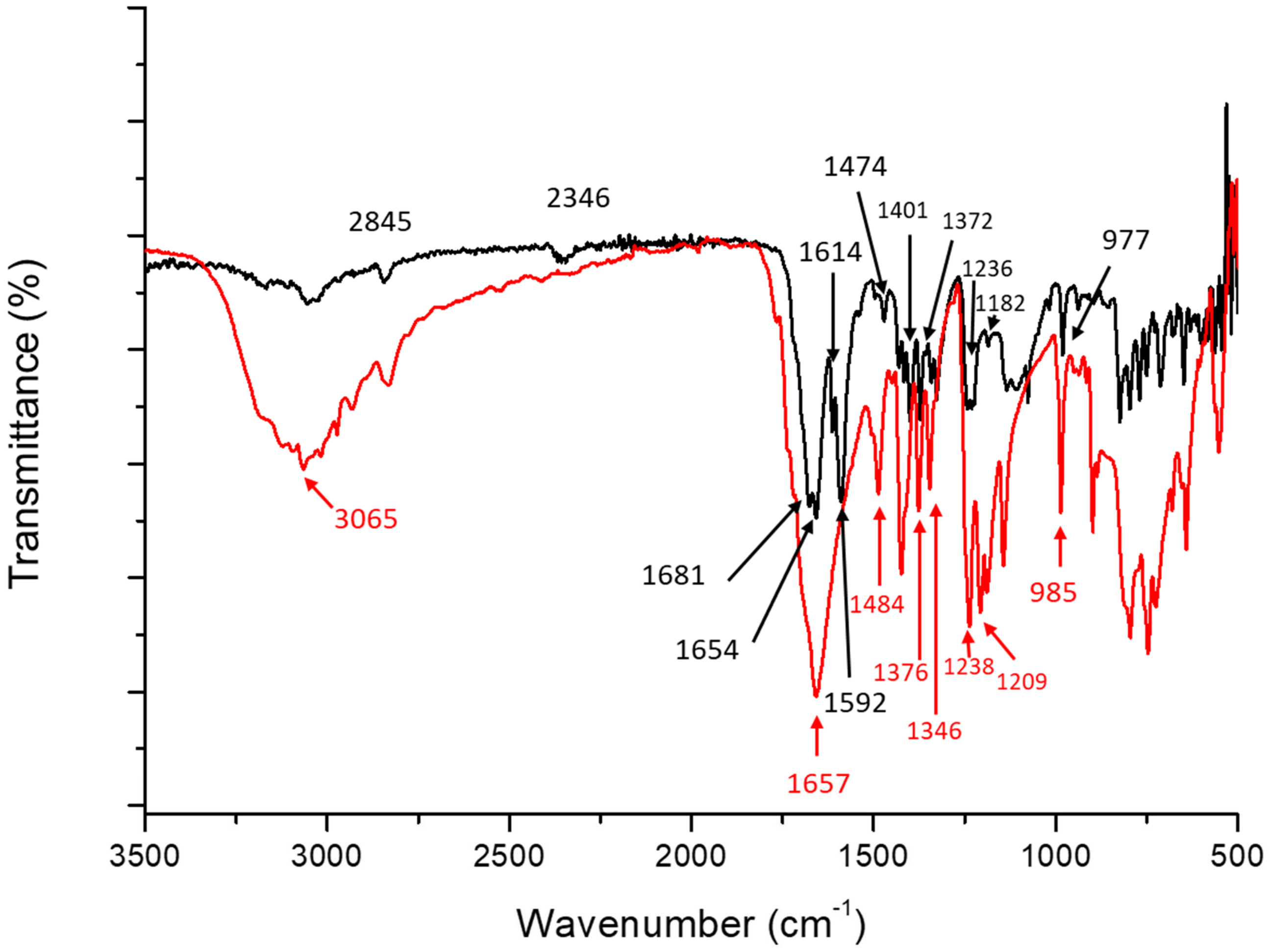

Figure S5. IR Spectrum of **[Cu$_2$(5-FUA)$_2$(ox)(bipy)]$_n$·2n H$_2$O (CP2**) black lin**e** and the ligan 5-FUA red line **.**

S2.2 Powder X-ray diffractograms.

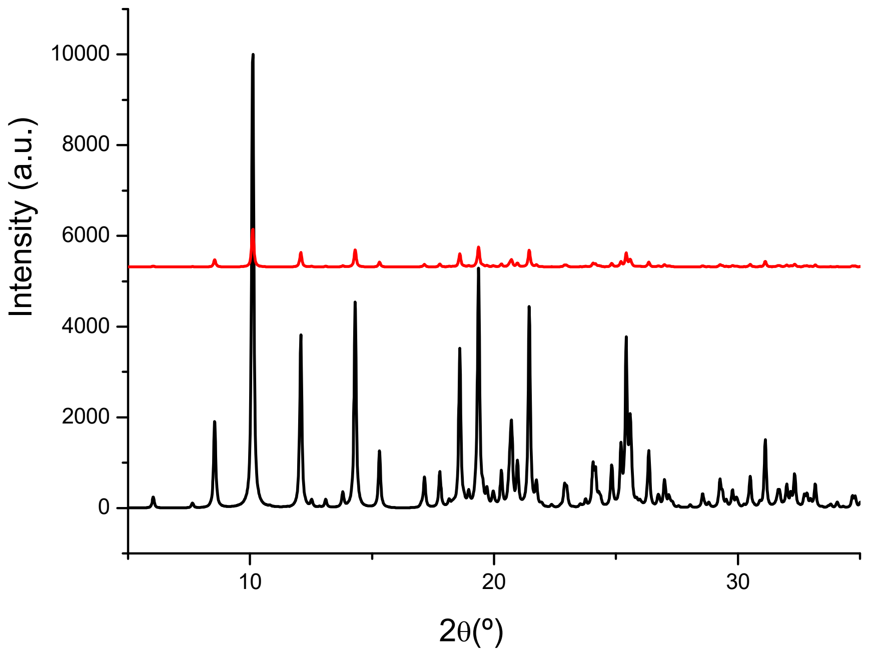

Figure S6. X-ray diffractogram of **$[Cu_2(5\text{-}FUA)_2(ox)(bipy)]_n \cdot 2n\ H_2O$ (CP2)**: Theoretical (black line) and experimental (red line).

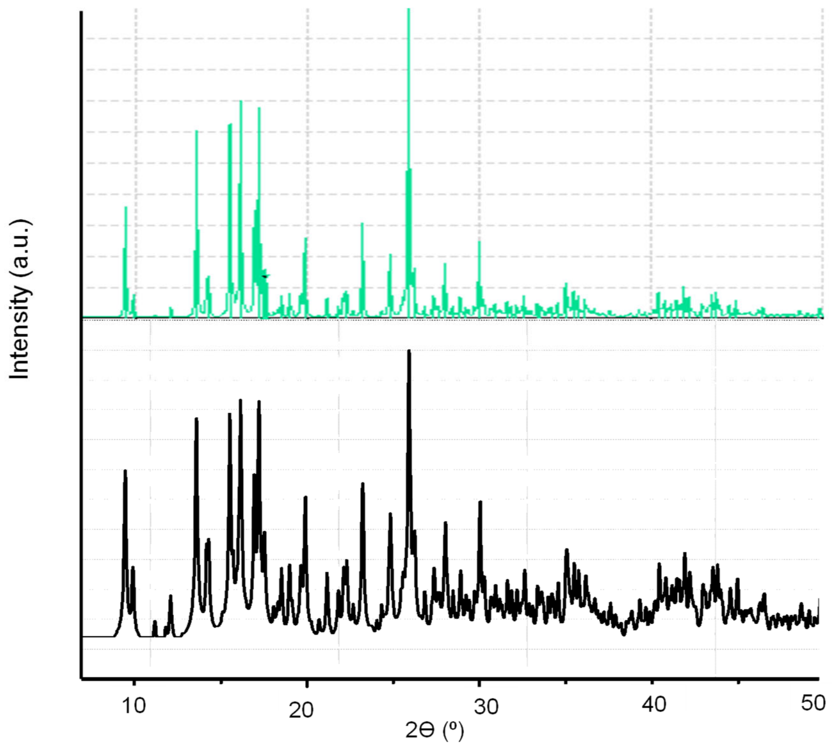

Figure S7. X-ray diffractogram of **[Cu(5-FUA)$_2$(H_2O)(bipy)]n 2n H_2O (CP3)**: Theoretical (black line) and experimental (green line).

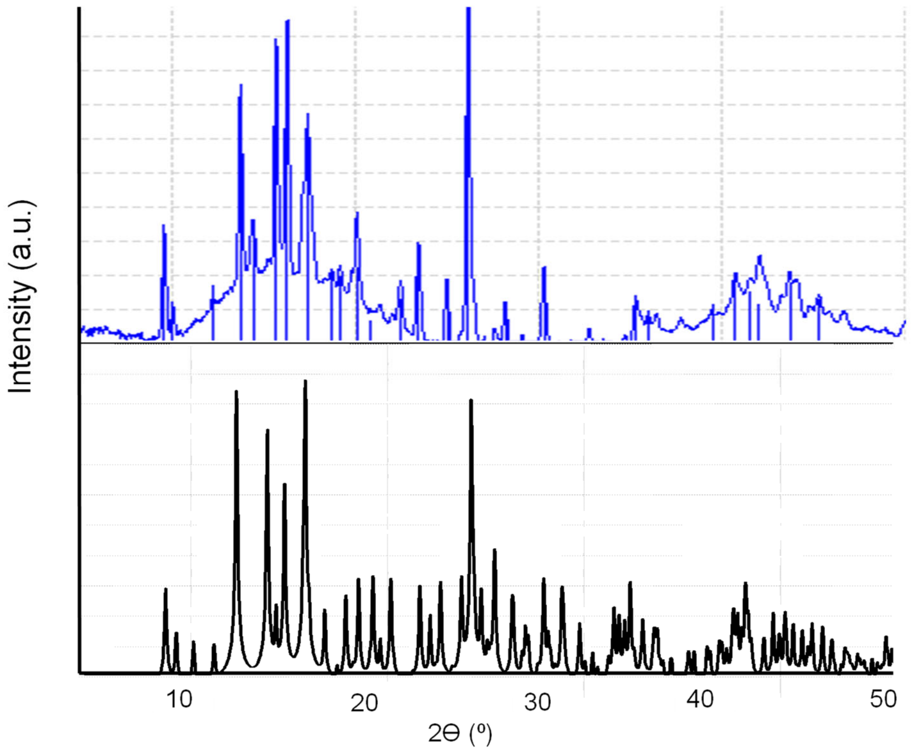

Figure S8. X-ray diffractogram of **[Cu(5-FUA)$_2$(bipy)]n 2n H_2O (CP4):** Theoretical (black line) and experimental (blue line).

S3. Crystal structure data of the compounds.

a. Compound [$(H_2bipy)^{+2}$ 2 NO_3^-] (1, CCDC 2243124)

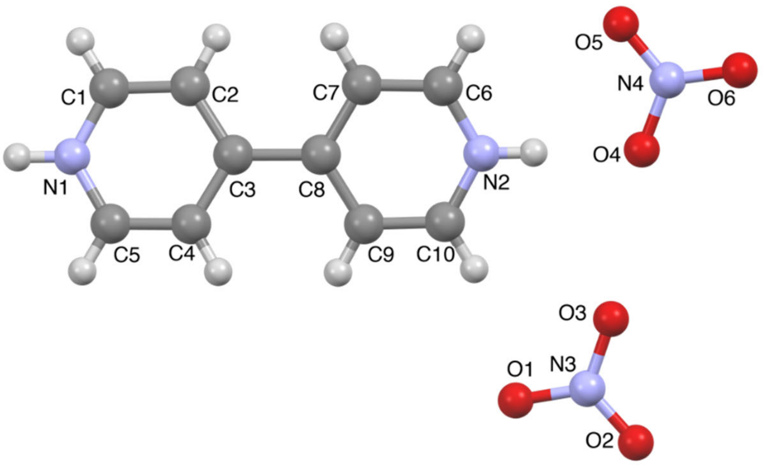

Figure S9. Asymmetric unit of compound **1** with non-hydrogen atoms labelled.

Table S1. Sample and crystal data for [$(H_2bipy)^{+2}$ 2 NO_3^-]

**Identification code**	**CCDC 2243124**	
**Chemical formula**	$C_{10}H_{10}N_4O_6$	
**Formula weight**	282.22 g/mol	
**Temperature**	250(2) K	
**Wavelength**	0.71073 Å	
**Crystal size**	0.063 x 0.124 x 0.131 mm	
**Crystal habit**	clear intense yellow prismatic	
**Crystal system**	monoclinic	
**Space group**	$P2_1/c$	
	a = 13.240(3) Å	α = 90°
**Unit cell dimensions**	b = 12.094(3) Å	β = 92.485(10)°
	c = 7.3852(18) Å	γ = 90°
**Volume**	1181.4(5) $Å^3$	
**Z**	4	
**Density (calculated)**	1.587 g/cm^3	
**Absorption coefficient**	0.134 mm^{-1}	
**F(000)**	584	

Table S2. Data collection and structure refinement for 1

**Theta range for data collection**	1.54 to 25.35°	
**Index ranges**	-15<=h<=15, -14<=k<=14, -8<=l<=8	
**Reflections collected**	18238	
**Independent reflections**	2158 [R(int) = 0.0727]	
**Coverage of independent reflections**	100.0%	
**Absorption correction**	Multi-Scan	
**Max. and min. transmission**	0.9920 and 0.9830	
**Structure solution technique**	direct methods	
**Structure solution program**	XT, VERSION 2018/2	
**Refinement method**	Full-matrix least-squares on F^2	
**Refinement program**	SHELXL-2018/3 (Sheldrick, 2018)	
**Function minimized**	$\Sigma\, w(F_o^2 - F_c^2)^2$	
**Data / restraints / parameters**	2158 / 0 / 189	
**Goodness-of-fit on F^2**	1.013	
**Final R indices**	1247 data; $I>2\sigma(I)$	$R_1 = 0.0440$, $wR_2 = 0.0945$
	all data	$R_1 = 0.1029$, $wR_2 = 0.1176$
**Weighting scheme**	$w=1/[\sigma^2(F_o^2)+(0.0562P)^2+0.0034P]$ where $P=(F_o^2+2F_c^2)/3$	
**Largest diff. peak and hole**	0.179 and -0.253 eÅ^{-3}	
**R.M.S. deviation from mean**	0.047 eÅ^{-3}	

b. Compound $[Cu_2(5\text{-}FUA)_2(ox)(bipy)]_n \cdot 2n\ H_2O$ (CP2) (CCDC 2243125)

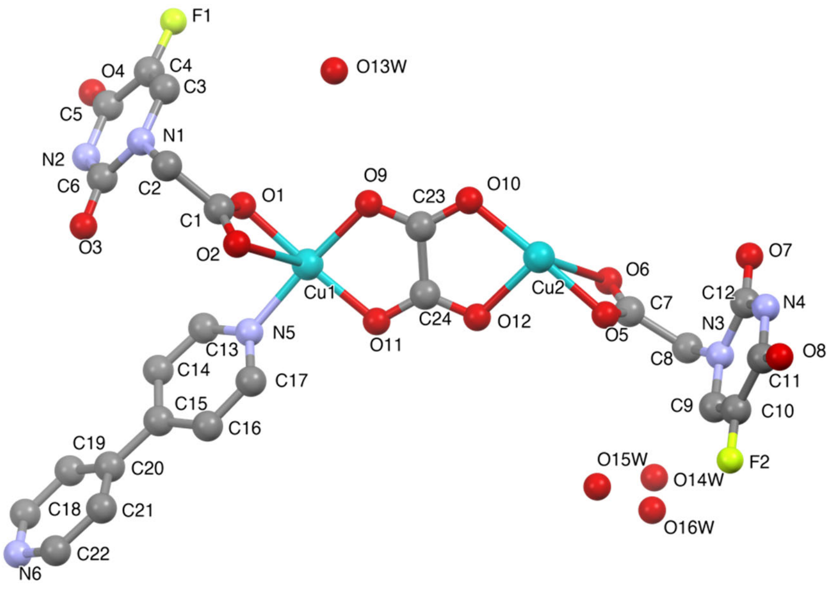

Figure S10. Asymmetric unit of compound $\mathbf{[Cu_2(5\text{-}FUA)_2(ox)(bipy)]_n \cdot 2n\ H_2O}$ (**CP2**) with atoms labelled. Hydrogen atoms have been omitted for clarity.

Table S3. Sample and crystal data for CP2

**Identification code**	**CCDC 2243125**	
**Chemical formula**	$C_{12}H_{8.38}CuFN_3O_7$	
**Formula weight**	389.13 g/mol	
**Temperature**	150(2) K	
**Wavelength**	0.71073 Å	
**Crystal size**	0.029 x 0.036 x 0.240 mm	
**Crystal habit**	clear blue needle	
**Crystal system**	triclinic	
**Space group**	*P*-1	
	a = 4.7345(2) Å	α = 85.8853(15)°
**Unit cell dimensions**	*b* = 14.7090(4) Å	β = 89.2707(16)°
	c = 20.7216(7) Å	γ = 86.4535(14)°
**Volume**	1436.52(9) Å^3	
**Z**	4	
**Density (calculated)**	1.799 g/cm^3	

**Absorption coefficient**	1.576 mm^{-1}
**F(000)**	782

Table S4. Data collection and structure refinement for CP2

**Theta range for data collection**	1.65 to 25.34°	
**Index ranges**	-5<=h<=5, -17<=k<=17, -24<=l<=24	
**Reflections collected**	49323	
**Independent reflections**	5236 [R(int) = 0.0470]	
**Coverage of independent reflections**	99.3%	
**Absorption correction**	multi-scan	
**Max. and min. transmission**	0.9560 and 0.7030	
**Structure solution technique**	direct methods	
**Structure solution program**	SHELXS-97 (Sheldrick 2008)	
**Refinement method**	Full-matrix least-squares on F^2	
**Refinement program**	SHELXL-2014/7 (Sheldrick, 2014)	
**Function minimized**	$\Sigma w(F_o^2 - F_c^2)^2$	
**Data / restraints / parameters**	5236 / 1 / 455	
**Goodness-of-fit on F^2**	1.035	
**Final R indices**	4274 data; I>2σ(I)	$R_1 = 0.0381$, $wR_2 = 0.0970$
	all data	$R_1 = 0.0516$, $wR_2 = 0.1046$
**Weighting scheme**	$w=1/[\sigma^2(F_o^2)+(0.0502P)^2+3.8042P]$ where $P=(F_o^2+2F_c^2)/3$	
**Largest diff. peak and hole**	1.463 and -0.439 eÅ^{-3}	
**R.M.S. deviation from mean**	0.098 eÅ^{-3}	

c. Compound $[Cu(5\text{-}FUA)_2(H_2O)(bipy)]_n \cdot 2n\ H_2O$ (CP3) (CCDC 2243126)

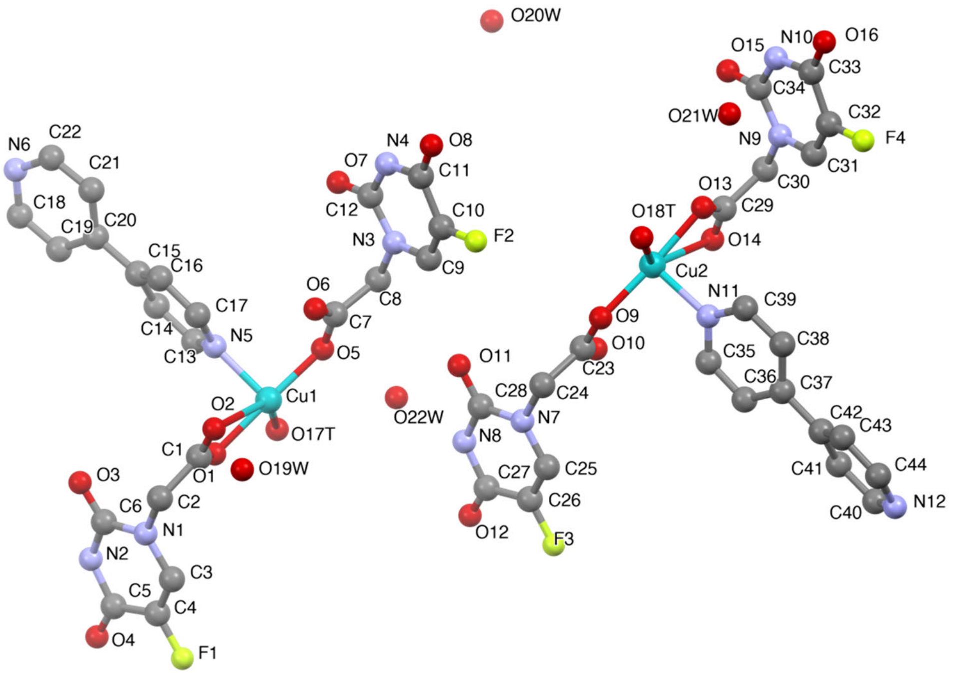

Figure S11. Asymmetric unit of compound **$[Cu(5\text{-}FUA)_2(H_2O)(bipy)]_n \cdot 2n\ H_2O$ (CP3)** with atoms labelled. Hydrogen atoms have been omitted for clarity.

Table S5. Sample and crystal data for CP3

**Identification code**	**CCDC 2243126**	
**Chemical formula**	$C_{22}H_{22}CuF_2N_6O_{11}$	
**Formula weight**	647.99 g/mol	
**Temperature**	200(2)	
**Wavelength**	0.71073 Å	
**Crystal size**	0.030 x 0.045 x 0.138 mm	
**Crystal habit**	clear intense blue prismatic	
**Crystal system**	monoclinic	
**Space group**	$P2_1/n$	
	a = 13.1870(7) Å	α = 90°
**Unit cell dimensions**	b = 21.9384(9) Å	β = 99.037(3)°
	c = 18.0621(9) Å	γ = 90°
**Volume**	5160.5(4) $Å^3$	
**Z**	8	
**Density (calculated)**	1.668 g/cm^3	

**Absorption coefficient**	0.934 mm^{-1}
**F(000)**	2648

Table S6. Data collection and structure refinement for CP3

**Theta range for data collection**	1.78 to 25.35°	
**Index ranges**	-15<=h<=15, -26<=k<=26, -21<=l<=21	
**Reflections collected**	43341	
**Independent reflections**	9450 [R(int) = 0.0815]	
**Coverage of independent reflections**	99.9%	
**Absorption correction**	multi-scan	
**Max. and min. transmission**	0.9730 and 0.8820	
**Refinement method**	Full-matrix least-squares on F^2	
**Refinement program**	SHELXL-2014/7 (Sheldrick, 2014)	
**Function minimized**	Σ w(F_o^2 - F_c^2)^2	
**Data / restraints / parameters**	9450 / 12 / 809	
**Goodness-of-fit on F^2**	1.005	
**Final R indices**	5883 data; I>2σ(I)	R_1 = 0.0471, wR_2 = 0.0921
	all data	R_1 = 0.0971, wR_2 = 0.1122
**Weighting scheme**	w=1/[$\sigma^2(F_o^2)$+(0.0451P)^2+0.0190P] where P=(F_o^2+2F_c^2)/3	
**Largest diff. peak and hole**	0.426 and -0.564 eÅ^{-3}	
**R.M.S. deviation from mean**	0.083 eÅ^{-3}	

d. Compound [Cu(5-FUA)$_2$(bipy)]$_n$·2n H_2O (CP4) (CCDC 2243127)

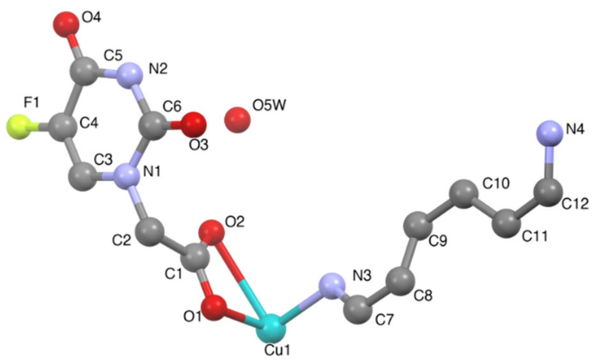

Figure S12. Asymmetric unit of compound **[Cu(5-FUA)$_2$(bipy)]$_n$·2n H_2O (CP4)** with atoms labelled. Hydrogen atoms have been omitted for clarity.

Table S7. Sample and crystal data for CP4

**Identification code**	**[Cu(5-FUA)$_2$(bipy)]$_n$·2n H_2O**	
**Chemical formula**	$C_{22}H_{20}CuF_2N_6O_{10}$	
**Formula weight**	629.98 g/mol	
**Temperature**	200(2) K	
**Wavelength**	0.71073 Å	
**Crystal size**	0.030 x 0.045 x 0.138 mm	
**Crystal habit**	clear violet plate	
**Crystal system**	monoclinic	
**Space group**	*C*2/*c*	
	a = 17.9239(10) Å	α = 90°
**Unit cell dimensions**	*b* = 11.0470(5) Å	β = 101.739(2)°
	c = 13.4050(7) Å	γ = 90°
**Volume**	2598.7(2) Å^3	
**Z**	4	
**Density (calculated)**	1.610 g/cm^3	
**Absorption coefficient**	0.922 mm^{-1}	
**F(000)**	1284	

Table S8. Data collection and structure refinement for CP4

**Theta range for data collection**	2.81 to 25.34°
**Index ranges**	-21<=h<=21, -12<=k<=12, -16<=l<=16

**Reflections collected**	23166	
**Independent reflections**	2356 [R(int) = 0.0379]	
**Max. and min. transmission**	0.9830 and 0.8090	
**Refinement method**	Full-matrix least-squares on F^2	
**Refinement program**	SHELXL-2019/1 (Sheldrick, 2019)	
**Function minimized**	$\Sigma w(F_o^2 - F_c^2)^2$	
**Data / restraints / parameters**	2356 / 0 / 199	
**Goodness-of-fit on F^2**	1.027	
**Final R indices**	2023 data; I>2σ(I)	R_1 = 0.0293, wR_2 = 0.0737
	all data	R_1 = 0.0381, wR_2 = 0.0787
**Weighting scheme**	$w=1/[\sigma^2(F_o^2)+(0.0397P)^2+3.7475P]$ where $P=(F_o^2+2F_c^2)/3$	
**Largest diff. peak and hole**	0.319 and -0.314 eÅ^{-3}	
**R.M.S. deviation from mean**	0.062 eÅ^{-3}	

e. Copper coordination environment parameters

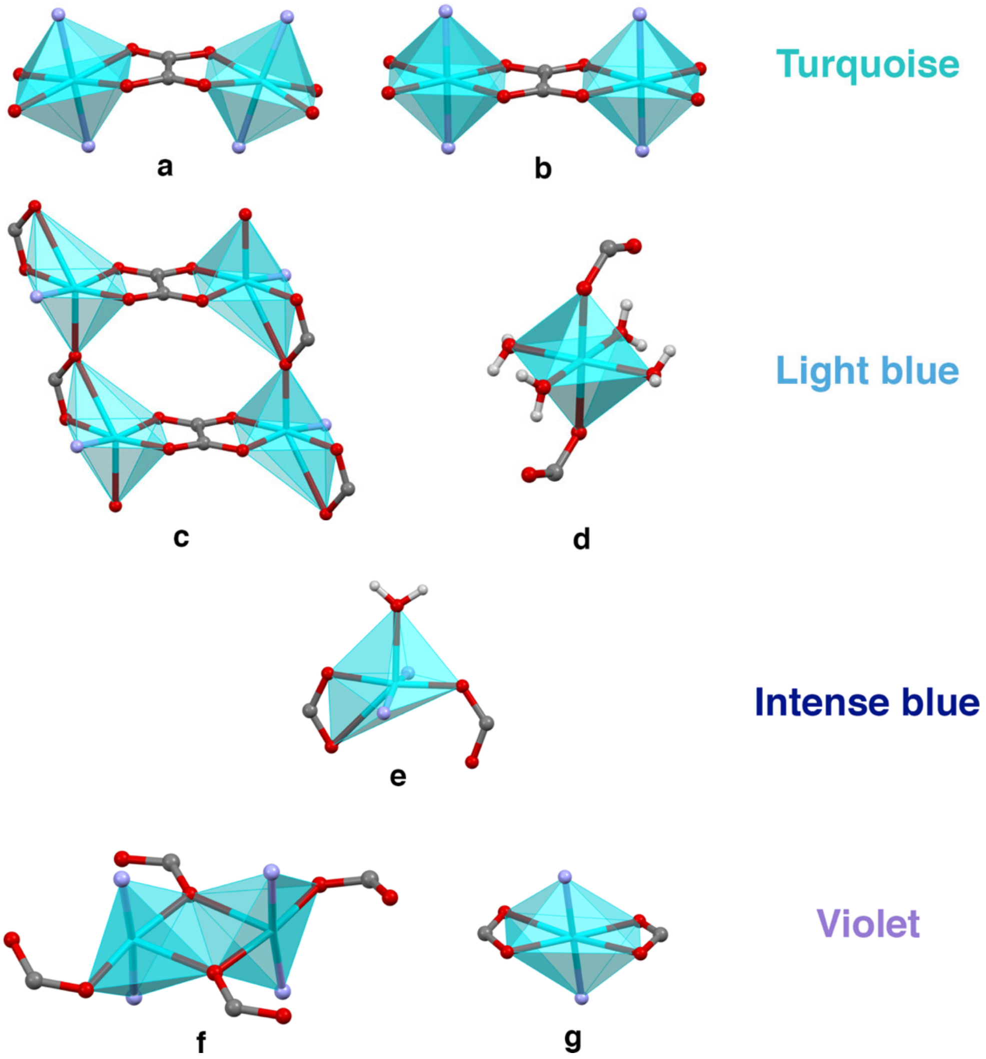

Figure S13. Coordination environments of copper atoms in the compounds with the resulting colours: **a)** pair of CuO_4N_2 octahedra from a zigzag row in **$[Cu_3(ox)_3(bipy)_4]_n$; b)** pair of **CuO_4N_2** octahedra from a linear row in **$[Cu(ox)(bipy)]_n$**; **c)** group of four CuO_5N polyhedra from a layer in **$[Cu_2(5\text{-}FUA)_2(ox)(bipy)]_n \cdot 2n\ H_2O$ (CP2); d)** CuO_6 octahedron in **$[Cu(5\text{-}FUA)_2(H_2O)_4] \cdot 4H_2O$; e)** CuO_4N_2 polyhedron in **$[Cu(5\text{-}FUA)_2(H_2O)(bipy)]_n \cdot 2n\ H_2O$ (CP3); f)** pair of edge-sharing square pyramids CuO_3N_2 in **$[Cu(5\text{-}FUA)_2(bipy)]_n \cdot 3.5n\ H_2O$**; **g)** highly distorted CuO_4N_2 octahedron in **$[Cu(5\text{-}FUA)_2(bipy)]_n \cdot 2n\ H_2O$ (CP4)**

Table S9 Selected coordination copper bond distances (Å) for compound **CP2**, **CP3** and **CP4.**

**$[Cu_2(5\text{-}FUA)_2(ox)(bipy)]_n..2nH_2O$ (CP2)**	Cu1-O1	1.945(2)
	Cu1-O2^{i}	2.184(2)
	Cu1-O2	2.756(2)
	Cu1-O9	1.998(2)
	Cu1-O11	1.972(2)
	Cu1-N5	1.983(3)
	Cu2-O5	1.945(2)
	Cu2-O6^{iii}	2.183(2)
	Cu2-O6	2.774(2)
	Cu2-O10	1.980(3)
	Cu2-O12	1.998(2)
	Cu1-N6^{ii}	1.978(3)
	Symmetry codes: (i) $x-1, y, z$; (ii) $x-1, y+1, z$; (iii) $x+1, y, z$	
**$[Cu(5\text{-}FUA)_2(H_2O)(bipy)]_n\cdot 2nH_2O$ (CP3)**	Cu1-O1	1.986(2)
	Cu1-O2	2.763(3)
	Cu1-O5	1.959(2)
	Cu1-O17T	2.305(3)
	Cu1-N5	2.005(3)
	Cu1-N6^{i}	2.009(3)
	Cu2-O9	1.957(2)
	Cu2-O13	1.984(2)
	Cu2-O14	2.738(3)
	Cu-O18T	2.341(3)
	Cu2-N11	2.012(3)
	Cu2-N12^{ii}	2.005(3)
	Symmetry codes: (i) $-x+1/2, y-1/2, -z+3/2$; (ii) $-x+1/2, y+1/2, -z+1/2$	
**$[Cu(5\text{-}FUA)_2(bipy)]_n\cdot 2nH_2O$ (CP4)**	Cu1-O1	1.979 (2)
	Cu1-O1^{i}	1.979 (2)
	Cu1-O2	2.599 (3)
	Cu1-O2^{i}	2.599 (3)
	Cu1-N3	1.987 (2)
	Cu1-N4^{ii}	1.995 (2)

	Symmetry codes: (i) −*x*+1, *y*, −*z*+1/2; (ii) *x*, *y*+1, *z*

Table S10. Selected copper coordination bond angles (°) for compounds **CP2**, **CP3** and **CP4**.

**[Cu$_2$(5-FUA)$_2$(ox)(bipy)]$_n$..2nH$_2$O (CP2)**	O1-Cu1-O2	52.74(9)
	O1-Cu1-O2^{i}	93.9(1)
	O1-Cu1-O9	91.2(1)
	O1-Cu1-O11	171.9(1)
	O1-Cu1-N5	92.1(1)
	O2-Cu1-O2^{i}	146.62(9)
	O2-Cu1-O9	90.97(9)
	O2-Cu1-O11	120.67(9)
	O2-Cu1-N5	81.5(1)
	O2^{i}-Cu1-O9	91.8 (1)
	O2^{i}-Cu1-O11	92.7(1)
	O2^{i}-Cu1-N5	99.9(1)
	O9-Cu1-O11	84.0(1)
	O9-Cu1-N5	167.6(1)
	O11-Cu1-N5	91.3(1)
	O5-Cu2-O6	52.24(9)
	O5-Cu2-O6^{iii}	93.1(1)
	O5-Cu2-O10	174.0(1)
	O5-Cu2-O12	92.9(1)
	O5-Cu2-N6^{ii}	90.3(1)
	O6-Cu2-O6^{iii}	145.30(8)
	O6-Cu2-O10	89.98(9)
	O6-Cu2-O12	122.78(9)
	O6-Cu2-N6^{ii}	81.1(1)
	O6^{iii}-Cu2-O10	91.6 (1)
	O6^{iii}-Cu2-O12	89.7(1)
	O6^{iii}-Cu2-N6^{ii}	103.9(1)
	O10-Cu2-O12	83.5(1)
	O10-Cu2-N6^{ii}	92.1(1)
	O12-Cu2-N6^{ii}	165.8(1)

	Symmetry codes: (i) *x*−1, *y*, *z*; (ii) *x*−1, *y*+1, *z;* (iii) *x*+1, *y*, *z*	
**[Cu(5-FUA)$_2$(H$_2$O)(bipy)]$_n$·2nH$_2$O (CP3)**	O1-Cu1-O2	52.86(9)
	O1-Cu1-O5	174.4(1)
	O1-Cu1-O17T	83.9(1)
	O1-Cu1-N5	90.4(1)
	O1-Cu1-N6^{i}	90.0(1)
	O2-Cu1-O5	132.76(9)
	O2-Cu1-O17T	138.48(9)
	O2-Cu1-N5	88.0(1)
	O2-Cu1-N6^{i}	82.2(1)
	O5-Cu1-O17T	90.5(1)
	O5-Cu1-N5	90.1(1)
	O5-Cu1-N6^{i}	90.8(1)
	O17T -Cu1-N5	98.2(1)
	O17T -Cu1-N6^{i}	95.7(1)
	N5-Cu1-N6^{i}	166.1(1)
	O13-Cu2-O14	53.08(9)
	O13-Cu2-O9	172.4(1)
	O13-Cu2-O18T	84.4(1)
	O13-Cu2-N11	92.1(1)
	O13-Cu2-N12^{ii}	90.3(1)
	O14-Cu2-O9	134.53(9)
	O14-Cu2-O18T	137.40(9)
	O14-Cu2-N11	84.8(1)
	O14-Cu2-N12^{ii}	84.4(1)
	O9-Cu2-O18T	88.0(1)
	O9-Cu2-N11	89.6(1)
	O9-Cu2-N12^{ii}	90.0(1)
	O18T -Cu2-N11	95.4(1)
	O18T -Cu2-N12^{ii}	100.2(1)
	N11 -Cu2- N12^{ii}	164.4(1)
	Symmetry codes: (i) −*x*+1/2, *y* −1/2, −*z*+3/2; (ii) −*x*+1/2, *y* +1/2, −*z*+1/2	
**[Cu(5-FUA)$_2$(bipy)]$_n$·2nH$_2$O (CP4)**	O1-Cu1-O1^{i}	178.59(9)
	O1-Cu1-O2	55.72(4)
	O1-Cu1-O2^{i}	124.2(2)
	O1-Cu1-N3	89.29(4)

	O1-Cu1-N4^{ii}	90.71(4)
	O1^{i}-Cu1-O2	124.2(2)
	O1^{i}-Cu1-O2^{i}	55.72(4)
	O1^{i} -Cu1-N3	89.29(4)
	O1^{i} -Cu1-N4^{ii}	90.71(4)
	O2-Cu1-O2^{i}	173.53(9)
	O2-Cu1-N3	86.77(4)
	O2-Cu1-N4^{ii}	93.23(4)
	O2^{i}-Cu1-N3	86.77(4)
	O2^{i}-Cu1-N4^{ii}	93.23(4)
	N3-Cu1-N4^{ii}	180
	Symmetry codes: (i) −*x*+1, *y*, −*z*+1/2; (ii) *x*, *y*+1, *z*	

f. Supramolecular interactions

Table S11. Relevant supramolecular hydrogen bond interactions found in compounds **CP2, CP3** and **CP4.** Interactions involving interstitial water molecules in blue, and the strongest ones in **bold**.

		D-H (Å)	A···H (Å)	D····A (Å)	D-H···A (°)
	C2-H2A···O1^{iii}	0.99	2.47	3.043(4)	117
	C3-H3···O13W^{v}	0.95	2.52	3.411(5)	157
	C8-H8A···O14W^{i}	0.99	2.23	3.221(8)	177
	C8-H8B···O5^{i}	0.99	2.4	3.089(4)	126
	C9-H9···O15W	0.95	2.41	3.224(10)	143
	C9-H9···O16W	0.95	2.27	3.193(17)	165
	C14-H14···O4^{iv}	0.95	2.25	3.145(4)	157
**$[Cu_2(5\text{-}FUA)_2(ox)(bipy)]_n \cdot 2nH_2O$ (CP2)**	C16-H16···F2^{vii}	0.95	2.6	3.227(4)	124
	C16-H16···O8^{vii}	0.95	2.55	3.492(5)	170
	C19-H19···O4^{vi}	0.95	2.4	3.345(4)	172
	C21-H21···O8^{vii}	0.95	2.57	3.337(4)	138
	C22-H22···O7^{viii}	0.95	2.37	3.239(4)	152
	**N2-H2N···O3^{ix}**	**0.88(4)**	**1.98(4)**	**2.854(4)**	**175(4)**
	**N4-H4N···O8^{x}**	**0.95(4)**	**1.91(4)**	**2.847(4)**	**170(4)**

	**O13W-H13A···O13W^{v}**	**0.81(2)**	**2.29(6)**	**2.827(9)**	**125(6)**

Symmetry codes: (i) x−1, y, z; (iii) x+1, y, z; (v) x+2, −y+1, −z+2; (vi) −x+2, −y+1, −z+2; (vii) −x+2, −y+1, −z+1; (viii) x+2, y−1, z; (ix) −x+2, −y, −z+2; (x) −x+1, −y+2, −z+1.

**[Cu(5-FUA)$_2$(H$_2$O)(bipy)]$_n$·2nH$_2$O (CP3)**	C2—H2A···O15^{v}	0.99	2.49	3.255 (5)	134
	**C3—H3A···O15^{v}**	**0.95**	**2.17**	**3.053 (5)**	**154**
	C8—H8A···O11	0.99	2.45	3.211 (4)	133
	**C9—H9···O11**	**0.95**	**2.2**	**3.082 (5)**	**153**
	C13—H13···O17T	0.95	2.52	3.203 (5)	128
	C16—H16···O4^{vi}	0.95	2.52	3.193 (5)	128
	C18—H18···O6^{iii}	0.95	2.53	3.366 (4)	147
	C19—H19···O8^{vii}	0.95	2.52	3.429 (5)	160
	C21—H21···O4^{vi}	0.95	2.33	3.271 (5)	171
	C24—H24A···O3^{i}	0.99	2.49	3.284 (4)	137
	**C25—H25···O3^{i}**	**0.95**	**2.18**	**3.065 (5)**	**154**
	C30—H30A···O4^{viii}	0.99	2.66	3.271(5)	121
	C30—H30A···O7^{iv}	0.99	2.53	3.211(5)	126
	C31—H31···O7^{iv}	0.95	2.25	3.129(5)	153
	C35—H35···O10	0.95	2.47	3.318(4)	149
	C36—H36···O16^{ix}	0.95	2.44	3.369 (5)	165
	**C38—H38···O12^{x}**	**0.95**	**2.3**	**3.235 (4)**	**170**
	C41—H41···O16^{ix}	0.95	2.54	3.066 (4)	115
	C43—H43···O12^{x}	0.95	2.47	3.107 (5)	124
	**N2—H2···O20W^{v}**	**0.84(4)**	**1.91(4)**	**2.739 (4)**	**169(4)**
	**N4—H4···O21W^{xi}**	**0.87(4)**	**1.97(4)**	**2.841 (4)**	**174(4)**
	**N8—H8···O22W**	**0.84(3)**	**1.89(4)**	**2.722 (4)**	**172(4)**
	**N10—H10···O19W^{xii}**	**0.92(4)**	**1.91(4)**	**2.817 (4)**	**174(4)**
	**O17T—H17A···O21W^{xiii}**	**0.90(2)**	**2.01(3)**	**2.805(4)**	**148(5)**
	**O17T—H17B···O10^{viii}**	**0.90(2)**	**1.98 (2)**	**2.853(4)**	**165(4)**
	**O18T—H18A···O2^{xiii}**	**0.87(2)**	**2.16(2)**	**3.002(4)**	**165(4)**
	**O18T—H18B···O6^{xiii}**	**0.88(2)**	**2.03(2)**	**2.876 (4)**	**163(5)**
	**O19W—H19A···O2**	**0.87(2)**	**1.92(2)**	**2.784(4)**	**172(4)**

	**O19W—H19B···O13^{xiii}**	**0.89(2)**	**2.07(2)**	**2.930(4)**	**164(4)**
	**O20W—H20A···O9^{ii}**	**0.89(2)**	**1.88(2)**	**2.771(4)**	**178(4)**
	**O20W—H20B···O6^{xiv}**	**0.88(2)**	**1.89(2)**	**2.760(4)**	**172(4)**
	**O21W—H21A···O1^{xiii}**	**0.87(2)**	**2.04(3)**	**2.825 (4)**	**151(4)**
	**O21W—H21B···O14^{xv}**	**0.89(2)**	**1.84(2)**	**2.689(4)**	**160(4)**
	**O22W—H22A···O5**	**0.88(2)**	**1.89(2)**	**2.758(4)**	**169(4)**
	**O22W—H22B···O10^{viii}**	**0.90(2)**	**1.88(2)**	**2.767(4)**	**173 (4)**
	Symmetry codes: (i) −x+1/2, y−1/2, −z+3/2; (ii) −x+1/2, y+1/2, −z+1/2; (iii) −x+1/2, y+1/2, −z+3/2; (iv) −x+1/2, y−1/2, −z+1/2; (v) x, y, z+1; (vi) x+1/2, −y+3/2, z−1/2; (vii) x−1/2, −y+3/2, z+1/2; (viii) −x, −y+1, −z+1; (ix) x−1/2, −y+1/2, z+1/2; (x) x+1/2, −y+1/2, z−1/2; (xi) −x+3/2, y+1/2, −z+1/2; (xii) x, y, z−1; (xiii) −x+1, −y+1, −z+1; (xiv) x−1/2, −y+3/2, z−1/2; (xv) x+1, y, z.				
**[Cu(5-FUA)$_2$(bipy)]$_n$·2nH$_2$O (CP4)**	C2-H2A···O3^{iv}	0.99	2.49	3.246(3)	133
	**C3-H3···O3^{iv}**	**0.95**	**2.31**	**3.181(3)**	**152**
	C8-H8···O4^{v}	0.95	2.42	3.111(3)	129
	C11-H11···O4^{v}	0.95	2.44	3.378(3)	170
	C12-H12···O2^{vi}	0.95	2.55	3.234(3)	129
	**N2-H2···O5W^{vii}**	**0.79(3)**	**1.98(3)**	**2.764(3)**	**178(3)**
	**O5W-H5WA···O2**	**0.72(3)**	**2.06(4)**	**2.775(3)**	**170(4)**
	**O5W-H5WB···O1^{viii}**	**0.74(4)**	**2.07(4)**	**2.779(3)**	**161(4)**
	Symmetry codes: (iv) -x+1/2, y+1/2, -z+1/2; (v) x+1/2, -y+3/2, z-1/2; (vi) -x+1, y -1, -z+1/2 ; (vii) -x+1/2, -y+3/2, -z+1; (viii) x, -y+2, -z+1/2				

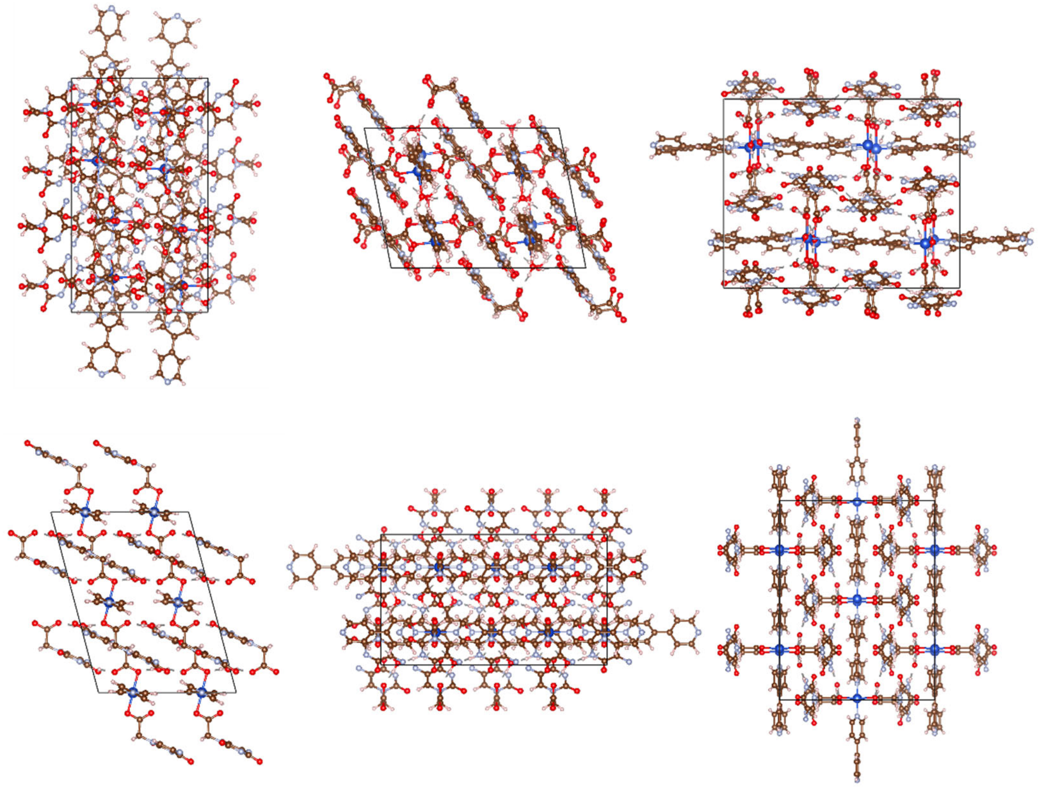

Figure S14. Optimized structures of compounds **[Cu(5-FUA)$_2$(H$_2$O)(bipy)]$_n$·2nH$_2$O** (**CP3**, top) and **[Cu(5-FUA)$_2$(bipy)]$_n$·2nH$_2$O** (**CP4**, bottom).
